# Performance of 6 HCV genotyping 9G test for HCV genotyping in clinical samples

**DOI:** 10.1186/s12985-018-1017-4

**Published:** 2018-07-11

**Authors:** Shrikant Dasharath Warkad, Satish Balasaheb Nimse, Keum-Soo Song, Wasun Chantratita, Viroj Pongthanapisith, Laxman Uddhav Nawale, Taisun Kim

**Affiliations:** 1 Institute for Applied Chemistry and Department of Chemistry, Hallym University, 1 Okcheon-dong, Chuncheon, 200-702 South Korea; 2 Department of Pathology, Faculty of Medicine, Virology Laboratory, Ramathibodi Hospital, Mahidol University, Bangkok, 10400 Thailand

**Keywords:** HCV, HCV genotyping, 9G DNA technology, INNO-LiPA, liver cirrhosis, hepatocellular carcinoma

## Abstract

**Background:**

A treatment of HCV infection depends on the genotype and sub-genotype. Therefore, accurate HCV genotyping is critical for selecting the appropriate treatment regimen.

**Method:**

This study included 280 plasma samples to evaluate the performance of 6 HCV Genotyping 9G test. The performance of 6 HCV Genotyping 9G test for accurate detection of HCV 1a, 1b, 2, 3, 4, and 6 genotypes was evaluated by comparing it with LiPA 2.0 assay and sequencing.

**Results:**

6 HCV Genotyping 9G test and LiPA 2.0 assay demonstrated 83.9% (*n* = 235) agreement. 39/45 samples that showed discrepant results between the two tests were analyzed by sequencing. Sequencing genotyped 39 discrepant samples as 0 (HCV 1a), 24 (HCV 1b), 1 (HCV 6f), 12 (HCV 6i), and 2 (HCV-negative). Results of 6 HCV Genotyping 9G test were very similar to the sequencing as it detected 1, 23, 1, 12, and 2 samples as HCV 1a, 1b, 3 & 6a or 6f, 6i or 6n, and negative, respectively. However, LiPA 2.0 assay showed complete disagreement with sequencing, as it did not detect any of these 39 samples correctly. These results indicate that LiPA 2.0 assay has limitations in identifying HCV genotypes 1b, and 6. The sensitivity, specificity, PPV, and NPV of 6 HCV Genotyping 9G test were 99.5, 98.8, 99.5, and 98.8%, respectively. It is important to note that HCV Genotyping 9G test showed 98.3 and 100% sensitivity for HCV 1b and 6 genotyping, respectively. However, LiPA 2.0 assay demonstrated 57.9 and 71.7% sensitivity for these genotypes.

**Conclusions:**

6 HCV Genotyping 9G test identifies HCV 1a, 1b, 2, 3, and 6 with good agreement with sequencing. Hence, 6 HCV Genotyping 9G test has a high clinical value because it can provide critical information to physicians and assist them to use the correct drug for efficient hepatitis C treatment.

**Electronic supplementary material:**

The online version of this article (10.1186/s12985-018-1017-4) contains supplementary material, which is available to authorized users.

## Background

Infection of hepatitis C virus (HCV) results in Hepatitis C, a liver disease. On a global scale, approximately 700,000 out of 150 million people with chronic HCV infection succumb each year to the hepatitis C-related liver diseases such as cirrhosis, hepatocellular carcinoma, and liver failure [1, 2]. Varieties in genotypes and subtypes of HCV complicated the treatment of HCV infection. Thus, the mortality attributable to HCV infection continues to increase [3]. There are 7 HCV genotypes and more than 90 subtypes with diverse patterns of geographic distribution. Globally, proportions of HCV genotypes G1, G3, G2, G4, G6, and G5 are 46.2, 30.1, 9.1, 8.3, 5.4, and 0.8%, respectively [4, 5]. Identification of the HCV genotype and sub-genotype is crucial for a proper antiviral treatment and cure of HCV-infected individuals [2, 6].

2016 World health organization guidelines for the screening, care, and treatment of chronic HCV infections recommend the identification of HCV genotypes to choose the precise treatment regimen [2]. These guidelines also recommend a nucleic acid test for HCV RNA to decide whether to start treatment for hepatitis C. The European Association for the Study of the Liver (EASL) endorsed that the treatment of HCV infections significantly depends on the genotypes and sub-genotype [7].

HCV infection is treated with the drugs such as pegylated interferon-α, ribavirin, and the direct-acting antivirals such as sofosbuvir, simeprevir, ledipasvir, ombitasvir, dasabuvir [8]. The use of monotherapy or multi-drug therapy and the duration of treatment critically depend on the HCV genotype [9]. Therefore, for accurate treatment, it is crucial to detect and discriminate the HCV genotypes 1a, 1b, 2, 3, 4, and 6 [10].

Currently, various nucleic acid tests such real-time PCR [11, 12], restriction fragment length polymorphism [13], heteroduplex mobility analysis [14], and line-probe assay [15] are used to detect HCV genotypes in plasma or serum. However, the agreement between the results of different methods is low [16, 17]. Therefore, sequence analysis of specific regions such as NS5, core, E1, and 5’UTR is considered as gold standard for HCV genotyping [18]. However, longer turn around time, cost, and requirement of highly trained professional to process the samples have limited the use of sequence analysis to the developed countries. Hence, there is a need for a rapid, simple, precise, and inexpensive genotyping test to execute the accurate treatment regime in the management of hepatitis C.

Recently, we have reported the 6 HCV Genotyping 9G test for the accurate detection of six HCV genotypes 1a, 1b, 2, 3, 4, and 6 in the plasma samples [19, 20]. The sensitivity and specificity of 6 HCV Genotyping 9G test were reported to be 100% for the sample containing 10 copies/test. However, only 63 HCV samples were used in the earlier study for the comparison of 6 HCV genotyping 9G test with VERSANT HCV genotype 2.0 assay (LiPA 2.0 assay) (Siemens Healthcare GmbH, Erlangen, Germany). The use of small sample size limits the clinical significance of the tests. It is important to note that the 6 HCV genotyping 9G test and LiPA 2.0 assay were not compared for HCV genotyping in blind clinical samples. Hence, in the present study, the performance of 6 HCV Genotyping 9G test is evaluated by comparing it with LiPA 2.0 assay for HCV genotyping in plasma samples of 280 individuals suspected of HCV infection. HCV was genotyped by sequence analysis in the samples that showed discordance between 6 HCV genotyping 9G test and LiPA 2.0 assay.

The workflow of 6 HCV Genotyping 9G test includes isolation of viral RNA, cDNA synthesis, PCR amplification, and detection of cyanine5.5 (Cy5) labeled PCR amplicons on the 9G membranes obtained by following the reported 9G technology [21]. The HCV genotype specific ssDNA oligonucleotide probes designed by following earlier report are immobilized on the 9G membranes at specific positions. The hybridization of Cy5 labeled PCR amplicons with immobilized probes allows the discrimination of six HCV genotypes. 6 HCV Genotyping 9G test genotypes HCV 1a, 1b, 2, 3, 4, 6a or 6f, and 6i or 6n at 25 °C in less than 30 min after PCR. Thus, the 6 HCV Genotyping 9G test can enable medical practitioners to follow EASL recommendations for accurate and highly effective hepatitis C management.

The HCV 1a and 1b account for almost 46.2% infections followed by HCV3 (30.1%), HCV2 301 (9.1%), HCV4 (8.3%), and HCV6 (5.4%) on a global scale. However, the HCV 6 accounts almost for 20% infections in the China and Southeast Asia. Hence, it is very crucial to discriminate these 303 genotypes correctly. Hence, the objective of this study was to evaluate the performance of 6 HCV Genotyping 9G test by comparing it with LiPA 2.0 assay and sequencing for accurate detection of HCV 1a, 1b, 2, 3, 4, and 6 genotypes.

## Methods

### Clinical samples

The documented prevalence rate of HCV in Thailand by several studies is in the range of 2.2 to 0.9% [22–24]. The sample size (n) was calculated by using formula n = [Z^2^ x P (1-P)/e^2^]d (http://www.who.int/ncds/surveillance/steps/resources/sampling/en/). The prevalence rate of HCV (1.6%) was used for sample size calculation, with the precision of 2% at 95% CI (Z = 1.96), a design effect (d = 1.5). The sample size was estimated to be 227, which was increased up to 280.

Plasma samples were collected from the 280 individuals including males and females (12–78 years, with the average age of 50 years) suspected of HCV infection. Consent to the use of samples from patients was obtained from the ethical committee. This study was approved by Ethical Clearance Committee on Human Rights Related to Research Involving Human Subjects, Faculty of Medicine Ramathibodi Hospital, Mahidol University. Samples were collected during the period of June 2015–June 2016 at the Mahidol University, Bangkok. Participants in this study were from various provinces of Thailand, and other countries such as Vietnam, Malaysia, Cambodia, Laos, and Myanmar.

The HCV RNA extraction was performed on the NucliSENS easyMAG (bioMérieux, Boxtel, Netherlands), that automatically extract the nucleic acids from the clinical samples. Plasma sample (200 μL) was mixed with lysis buffer and allowed to incubate for 10 min at room temperature. Magnetic silica particles were used for nucleic acid binding for 10 min at room temperature. Silica particles were washed with different buffers. HCV RNA was eluted in 50 μL of Tris-elution buffer and subsequently transcribed into cDNA by following the protocols of LiPA 2.0 assay and 6 HCV Genotyping 9G test.

### 6 HCV genotyping 9G test

6 HCV Genotyping 9G test is performed by adding 110 μL of hybridization solution (25% Formamide, 0.1% Triton X-100, 6× SSC) into the PCR tube containing 20 μL of PCR product. Then, 110 μL of this mixture was loaded into the sample port and allowed to stand for 20 min at 25 °C. After 20 min, 200 μL of washing solution (4× SSC) was loaded into the washing port and allowed to stand for 8 min at 25 °C. After 8 min, each 6 HCV Genotyping 9G test strip was scanned on the BMT Reader™ (Biometrix Technology Inc. Chuncheon, South Korea) and the results were automatically interpreted by the 9G Test™ Analyzer program. Results of the 6 HCV Genotyping 9G test are presented in Table 1, Table 2, and Table 3.

**Table 1:** Comparison of the genotyping results of 6 HCV Genotyping 9G test with LiPA 2.0 assay for plasma samples (*n* = 280)

HCV Genotype	No. of subtypes		*κ* value (95% CI)
	6 HCV Genotyping test	LiPA 2.0 assay	
1a	25	33	0.155 (0.119–0.429)
1b	60	43	0.077 (0.067–0.220)
2	3	3	1.000 (1.000–1.000)
3	60	61	0.659 (0.0367–1.000)
4	0	0	–
6	17 (6a or 6f), 30 (6i or 6n)	59 (6 (c-l))	0.127 (0.100–0.353)
3 & 6	2 (3 and 6i or 6n) 1 (3 and 6a or 6f)	2 (3 and 6(c-l))	0.500 (0.235–1.000)
Negative	82	80	0.491 (0.109–1.000)

**Table 2:** Agreement between the results of 6 HCV Genotyping 9G test and LiPA 2.0 assay (n = 235)

HCV Genotype	No. of subtypes	
	6 HCV Genotyping test	LiPA 2.0 assay
1a	22	22
1b	33	33
2	3	3
3	60	60
4	0	0
6	17 (6a or 6f), 18 (6i or 6n)	35 (6 (c-l))
3 & 6	2 (3&(6a or 6f))	2 (3& 6(c-l))
Negative	80	80

**Table 3:** Agreement between the results of sequencing, 6 HCV Genotyping 9G test, and LiPA 2.0 assay for discrepant samples (*n* = 39)

HCV Genotype	No. of subtypes		
	Sequencing	6 HCV Genotyping test	LiPA 2.0 assay
1a	0	0	0
1b	24^a,b^	23	0
2	0	0	0
3	0	0	0
4	0	0	0
6	1 (6f)^c^ 12 (6i)^d^	1 (3 & 6a or 6f) 12 (6i or 6n)	0
Negative	2^e^	2	0

^a^6 HCV Genotyping test detected 1 sample as 1a; LiPA 2.0 assay genotyped 39 samples as, ^b^6 samples as HCV1a and 18 samples as HCV 6(c-l), ^c^1 sample as HCV 3, ^d^2 samples HCV 1a and 10 samples as HCV 1b, ^e^2 samples as HCV 6(c-l)

### LiPA 2.0 assay

The viral RNA was purified using a QIAamp viral RNA minikit (Qiagen, Hilden, Germany) and subjected to reverse transcription-PCR (RT-PCR) with a Versant HCV amplification 2.0 kit (manufactured by Innogenetics, Ghent, Belgium, for Siemens, Tarrytown, NY, USA). HCV genotypes were detected in 280 plasma samples by following the manufacturers’ protocol. In brief, the 240-bp 5’UTR and 270-bp core fragments were co-amplified to produce biotinylated PCR product. Biotinylated PCR products were hybridized to the immobilized oligonucleotide probes that are specific for the 5’-UTRs and core regions of the six HCV genotypes. The hybridized biotinylated PCR products were detected by using alkaline phosphatase-labeled streptavidin and 5-bromo-4-chloro-3-indolylphosphate (BCIP)-p-nitroblue tetrazolium chromogen. Results were interpreted according to the LiPA 2.0 assay interpretation chart, where the line patterns and the corresponding genotyping results are listed. Results of the LiPA 2.0 test are shown in Table 1, Table 2, and Table 3.

### Reference HCV genotyping

The sequence of amplified fragment (from clinical samples) was determined on Applied Biosystems (ABI) 3730XL DNA analyzer (Life Technologies Co., Carlsbad, CA, USA). The specific HCV genotypes were confirmed by comparing the obtained sequences with the reported sequences on the Basic Local Alignment Search Tool (BLAST) database of national center of biotechnology information (NCBI). Sequencing was not performed for 6/46 discordant samples because sufficient amount of sample was unavailable. Results of the sequencing are arranged in Table 3.

### Statistical analysis

The *k* coefficient was used to assess consistency between 6 HCV Genotyping 9G test and LiPA 2.0 assay. The *k* values between 0.61 and 0.80 indicate good agreement between the two tests. The sensitivity, specificity, positive predictive (PPV) and negative predictive values (NPV) at 95% confidence interval (CI) were calculated. Statistical analyses were performed by using the statistical program Medcalc for Windows version 17.4.4 (Medcalc Software, Mariakerke, Belgium). A result was considered as true positive (TP) if the 6 HCV Genotyping 9G test, LiPA 2.0 assay, and sequencing (in the case of discordant samples) showed the same HCV genotype. A result was considered as true negative (TN) if the 6 HCV Genotyping 9G test, LiPA 2.0 assay, and sequencing (in the case of discordant samples) did not detect any HCV genotype in the sample.

In 45 discordant samples, results of 6 HCV Genotyping 9G test and LiPA 2.0 were considered as false positive (FP) if the number of a particular HCV genotype identified exceeds the number of that genotype detected by sequencing. Results of 6 HCV Genotyping 9G test and LiPA 2.0 were considered as false negative (FN) if the number of a particular HCV genotype detected is less than the number of that genotype detected by sequencing. Test results were classified as TP, TN, FP, and FN. From these categories, sensitivity (TP/TP + FN), specificity (TN/TN + FP), PPV (TP/TP + FP) and NPV (TN/FN + TN) values were calculated with 95% confidence intervals (CI).

## Results

As shown in Tables 1, 6 HCV Genotyping test detected 198 and 82 samples as HCV positive and HCV negative, respectively. Out of 198 HCV positive samples, 6 HCV Genotyping test detected 25 (12.6%), 60 (30.3%), 3 (1.51%), 60 (30.3%), 17 (8.59%), 30 (15.2%), samples as HCV genotypes 1a, 1b, 2, 3, 6a or 6f, and 6i or 6n, respectively. The prevalence of HCV genotypes detected by 6 HCV Genotyping test is similar to the recently reported prevalence of HCV genotypes in Thailand [25]. In three samples containing mixed-genotypes, 2 and 1 samples were identified as 3 & 6i or 6n, 3 & 6a or 6f, respectively by 6 HCV Genotyping test. As shown in Table 1, LiPA 2.0 assay detected 200 samples HCV positive and 80 samples as HCV negative. Out of 200 HCV positive samples, LiPA 2.0 assay genotyped 33 (16.5%), 43 (21.5%), 3 (1.50%), 61 (30.5%), 59 (29.0%), and 2 (1.00%) samples as HCV 1a, 1b, 2, 3, 6(c-l), and 3 & 6(c-l), respectively.

Agreement rate between the 6 HCV Genotyping 9G test and LiPA 2.0 assay was assessed by calculating *k* (95% CV) values for each HCV genotype. The *k* (95% CV) values were in the range of 0.077 (0.067–0.220) - 0.659 (0.109–1.000). It is important to note that 6 HCV Genotyping 9G test and LiPA 2.0 assay demonstrated good agreement for the detection of HCV 2 and 3. However, these results indicate that the 6 HCV Genotyping 9G test and LiPA 2.0 assay had a very poor agreement in the detection of important HCV genotypes such as HCV 1a, 1b and 6. Hence, the data was analyzed at an individual sample level, which indicated the 6 HCV Genotyping 9G test and LiPA 2.0 assay showed agreement in 235/280 samples as depicted in Table 2.

In 235 samples, 6 HCV Genotyping 9G test and LiPA 2.0 assay detected 22, 33, 3, 60, 35, and 80 as HCV 1a, 1b, 2, 3, 6, and negative, respectively. Out of 35 HCV 6 samples, 6 HCV Genotyping 9G test detected 17 and 18 samples as HCV (6a or 6f) and HCV (6i or 6n), respectively. Results of 45/280 samples were found to have a disagreement between two methods. Consequently, 6 HCV Genotyping 9G test was found to have only 83.9% of agreement with LiPA 2.0 assay.

Therefore, to determine the accuracy of the 6 HCV Genotyping 9G test and performance against LiPA 2.0 assay, the discrepant samples were genotyped by sequencing as presented in Table 3 (See the Additional file 1: Table S1). Out of 45 discrepant samples, sequencing was not performed on the six samples due to unavailability of remnant nucleic acid. Out of remaining 39 discrepant samples, sequencing genotyped 0, 24, 1, 12, and 2 samples as HCV 1a, 1b, 6f, 6i, and negative, respectively. Results of 6 HCV Genotyping 9G test were similar to the sequencing as it detected 1, 23, 1, 12, and 2 samples as HCV 1a, 1b, 3 & 6a or 6f, 6i or 6n, and negative, respectively. However, as depicted in Table 3, LiPA 2.0 assay showed complete discrepancy with sequencing, as it did not detect any of these 39 samples correctly (See Additional file 1: Table S1). 6 HCV Genotyping test showed one FP result as HCV 1a and 1/24 FN result for HCV 1b sample. Out of 24 HCV 1b samples genotyped by sequencing, LiPA 2.0 assay detected 6 samples as HCV 1a and 18 samples as HCV 6(c-l). A sample detected as HCV 6f in sequencing, LiPA 2.0 assay detected it as HCV 3. Out of 12 HCV 6i samples identified by sequencing, LiPA 2.0 assay genotyped 2 samples HCV 1a and 10 samples as HCV 1b. Two negative samples in sequencing were detected as HCV 6(c-l) by LiPA 2.0 assay. Hence, LiPA 2.0 assay showed complete dis-agreement with sequencing in 39 samples.

In case of six samples that did not have sequencing data, 6 HCV Genotyping 9G test genotyped 2 and 4 samples as HCV 1a and 1b, respectively. LiPA 2.0 assay detected three samples each as HCV 1a and 6(c-l). However, there was no agreement between 6 HCV Genotyping 9G test and LiPA 2.0 assay for genotyping of these six samples. Hence, these six samples were not used to determine the accuracy of 6 HCV Genotyping 9G test and LiPA 2.0 assay. The other reason to exclude these samples was unavailability of sequencing data. As shown in Tables 2 and 3, 6 HCV Genotyping 9G test detected mixed HCV genotypes in three samples. However, LiPA 2.0 assay detected mixed HCV genotypes in two samples. It is important to note that sequencing did not detect mixed HCV genotypes in any of the samples. The samples that were identified to contain single genotype in sequencing but mixed genotype in 6 HCV Genotyping 9G test and LiPA 2.0 assay were considered as TP if one of the genotypes is same as identified by sequencing.

To determine the HCV genotype level sensitivity and specificity of 6 HCV Genotyping 9G Test and LiPA 2.0, the TP, TN, FP, and FN values were identified from Table 2 and Table 3 (See Additional file 1: Table S2). The calculated sensitivity and specificity were organized in Table 4. 6 HCV Genotyping 9G test and LiPA 2.0 demonstrated 100% sensitivity for genotyping HCV 1a, 2, 3, and negative samples. The specificity of 6 HCV Genotyping 9G test and LiPA 2.0 were 99.6 99.8% for genotyping HCV 1a. 6 HCV Genotyping 9G test and LiPA 2.0 demonstrated 100% sensitivity for genotyping HCV 2, 3, and negative samples. However, specificity of 6 HCV Genotyping 9G test and LiPA 2.0 assay for genotyping HCV negative samples was 100 and 97.6%, respectively. It is important to note that the sensitivity of 6 HCV Genotyping 9G test for genotyping of HCV 1b was 98.3%. Conversely, LiPA 2.0 assay showed only 57.9% sensitivity for genotyping of HCV 1b. The specificity of 6 HCV Genotyping 9G test and LiPA 2.0 assay for genotyping HCV 1b samples were 100 and 95.6%, respectively. Sensitivity and specificity of 6 HCV Genotyping 9G test for HCV 6 genotyping were 100%. However, for HCV 6 genotyping LiPA 2.0 assay showed only 72.9 and 99.2% of sensitivity and specificity, respectively. These results clearly indicate that 6 HCV Genotyping 9G test is superior over LiPA 2.0 assay for the genotyping of HCV 1a, 1b, and 6.

**Table 4:** Sensitivity and specificity of 6 HCV Genotyping 9G test and LiPA 2.0 assay for particular HCV genotype

HCV genotype	6 HCV Genotyping 9G test		LiPA 2.0 Assay	
	Sensitivity (95% CI)	Specificity (95% CI)	Sensitivity (95% CI)	Specificity (95% CI)
1a	100.0 (84.6–100.0)	99.6 (97.8–99.9)	100.0 (84.6–100.0)	96.8 (93.8–98.6)
1b	98.3 (90.6–99.9)	100.0 (98.3–100.0)	57.9 (44.1–70.9)	95.4 (91.7–97.8)
2	100.0 (29.2–100.0)	100.0 (98.7–100.0)	100.0 (29.2–100.0)	100.0 (98.6–100.0)
3	100.0 (94.1–100.0)	100.0 (98.3–100.0)	100.0 (94.0–100.0)	99.5 (97.4–100.0)
4	–	–	–	–
6	100.0 (92.6–100.0)	100.0 (98.4–100.0)	72.9 (58.2–84.7)	91.2 (86.7–94.5)
3&6	100.0 (15.8–100.0)	100.0 (98.7–100.0)	100.0 (15.8–100.0)	100.0 (98.7–100.0)
Negative	100.0 (98.1–100.0)	100.0 (95.6–100.0)	100.0 (98.1–100.0)	97.6 (91.5–99.7)

As shown in Table 5, the overall sensitivity, specificity, PPV, and NPV of 6 HCV Genotyping 9G test were determined (See Additional file 1: Table S3). The overall sensitivity and specificity of 6 HCV Genotyping 9G test were 99.5% (97.1–100.0% at 95% CI) and 98.8% (93.5–99.9% at 95% CI), respectively. The PPV and NPV of 6 HCV Genotyping 9G test were 99.5% (96.4–99.9% at 95% CI) and 98.8% (92.1–99.8% at 95% CI), respectively. These results showed that 6 HCV Genotyping 9G test is a highly accurate as it has high agreement rate with sequencing analysis. Moreover, the PPV and NPV results of clinical samples (*n* = 274) indicate that 6 HCV Genotyping 9G test is a highly efficient method for the correct genotyping of six HCV genotypes.

**Table 5:** Overall sensitivity, specificity PPV, and NPV of 6 HCV Genotyping 9G Test for HCV genotyping (n = 274)

Sensitivity (95% CI)	Specificity (95% CI)	PPV (95% CI)	NPV (95% CI)
99.5 (97.1–100.0)	98.8 (93.5–99.9)	99.5 (96.4–99.9)	98.8 (92.1–99.8)

## Discussion

HCV is curable if the proper drug regimen is chosen to target a specific HCV genotype. Accurate identification of the HCV genotypes in the HCV-infected patients allows physicians to use correct drug regimen recommended by EASL. Therefore, it is imperative for clinical laboratories to use the most accurate HCV genotyping method to provide in-depth information on HCV genotype to clinicians for better patient care. It is important to note that 6 HCV Genotyping 9G test demonstrated high accuracy in the detection of HCV genotypes in plasma samples obtained from 280 individuals.

The applications of novel diagnostic platforms are usually limited due to the absence of an extensively sensitive standard for comparison. The literature on HCV genotyping indicates that the agreement between the results of any two HCV genotyping results is poor. Hence, a method that can be as accurate as sequencing but simple to use is required for efficient HCV genotyping. In this study, 6 HCV Genotyping 9G test was compared with the commercial LiPA 2.0 assay, which is also a line probe assay. The sequencing analysis was used as the standards for the detection of the HCV genotypes in 46 clinical samples under blinded codes.

The 6 HCV Genotyping 9G test showed very good agreement with the results of sequencing. However, LiPA 2.0 assay showed many discrepancies with the results of sequencing. The sensitivity of LiPA 2.0 assay for the detection of HCV 1b and 6 were only 57.9 and 71.7%. It is important to note that the HCV 1b and 6 are the most predominant genotypes in the Asian countries. Hence, the accurate detection of these genotypes is crucial for HCV therapy. It is reported earlier that LiPA 2.0 assay has limitations for the correct identification of HCV 1a, 1b, and HCV 6 [26]. The HCV genotypes and subtypes have more than 95% of the sequence homology. Thus, a likely reason for the discrepancies in the results of the sequencing and LiPA 2.0 assay can be the high percentage of sequence homology in HCV genotypes. A report on the comparison of Trugene assay and Lipa 2.0 assay with sequencing indicated that these tests failed to differentiate between HCV subtypes 1a and 1b [22]. Furthermore, the LiPA 2.0 assay and the Abbott Realtime HCV Genotype II assay were found to have limitations in identifying HCV genotype 6 [27]. A failure in correct HCV genotyping can lead to critical errors in clinical practice for choosing optimal drug therapy. 6 HCV Genotyping 9G test demonstrated very high sensitivity, specificity, PPV, and NPV for HCV genotyping. Hence, 6 HCV Genotyping 9G test can be effectively used in the clinical practice.

## Conclusion

To conclude, HCV Genotyping 9G test showed 98.3 and 100% sensitivity for HCV 1b and 6 genotyping, respectively. However, LiPA 2.0 assay demonstrated 57.9 and 71.7% sensitivity for these genotypes. Overall sensitivity and specificity of 6 HCV Genotyping 9G test were found to be 99.5% (97.1–100.0% at 95% CI) and 98.8% (93.5–99.9% at 95% CI), respectively. The 99.5% (96.4–99.9% at 95% CI) PPV and 98.8% (92.1–99.8% at 95% CI) NPV clearly indicate that 6 HCV Genotyping 9G test can correctly identify the HCV positive and negative samples. Hence, the results of this study indicate that 6 HCV Genotyping 9G test is a reliable, sensitive, and accurate diagnostic tool for HCV genotyping in the clinical samples, which is vital information for the choice of definitive drug therapy.

## Additional file

Additional file 1: **Table S1.** Comparison of 6 HCV Genotyping 9G Test and LiPA 2.0 with the sequencing in 46 discordant samples. **Table S2.** TP, TN, FP, and FN results of 6 HCV Genotyping 9G Test and LiPA 2.0 with the sequencing in 274 samples. **Table S3.** TP, TN, FP, and FN results of 6 HCV Genotyping 9G Test (*n* = 274). (DOCX 86 kb)

## Abbreviations

HCV: hepatitis C virus
HCV 1a: HCV genotype 1a
HCV 1b: HCV genotype 1b
HCV 2: HCV genotype 2
HCV 3: HCV genotype 3
HCV 4: HCV genotype 4
HCV 6: HCV genotype 6
EASL: European Association for the Study of the Liver
6 HCV Genotyping 9G test: six HCV genotyping 9G test
LiPA 2.0 assay: VERSANT HCV genotype 2.0 assay
Cy5: Cyanine5.5 dye
BLAST: Basic Local Alignment Search Tool
NCBI: national center of biotechnology information
PPV: positive predictive (PPV)
NPV: negative predictive values (NPV)
CI: confidence interval
TP: true positive
NT: true negative (TN)
