# Supplementary material for: Performance of 6 HCV genotyping 9G test for HCV genotyping in clinical samples
Source: Virol J. 2018 Jul 11;15:107. doi: 10.1186/s12985-018-1017-4 (PMC6042330; doi:10.1186/s12985-018-1017-4)
Supplement: Supplementary file 1 — Table S1. Comparison of 6 HCV Genotyping 9G Test and LiPA 2.0 with the sequencing in 46 discordant samples. Table S2. TP, TN, FP, and FN results of 6 HCV Genotyping 9G Test and LiPA 2.0 with the sequencing in 274 samples. Table S3. TP, TN, FP, and FN results of 6 HCV Genotyping 9G Test (n = 274). (DOCX 86 kb) [file 12985_2018_1017_MOESM1_ESM.docx]

**Additional file**

**Table S1:** Comparison of 6 HCV Genotyping 9G Test and LiPA 2.0 with the sequencing in 46 discordant samples

| **Sr. No.** | **Sample No.** | **Sequencing** | **6 HCV Genotyping 9G test** | **LiPA 2.0 Assay** | **Comparison with Sequencing** | |
| --- | --- | --- | --- | --- | --- | --- |
|  |  |  |  |  | **6 HCV Genotyping 9G test** | **LiPA 2.0 Assay** |
|  | 5 | 1b | 1b | 6 (c-l) | o | x |
|  | 12 | No data | 1b | 6 (c-l) | - | - |
|  | 13 | No data | 1a | 6 (c-l) | - | - |
|  | 14 | 1b | 1b | 1a | o | x |
|  | 19 | 1b | 1b | 6 (c-l) | o | x |
|  | 20 | 6i | 6i or 6n | 1b | o | x |
|  | 21 | 1b | 1b | 1a | o | x |
|  | 22 | 1b | 1b | 1a | o | x |
|  | 25 | negative | Negative | 6 (c-l) | o | x |
|  | 31 | 6i | 6i or 6n | 1b | o | x |
|  | 33 | 1b | 1b | 6 (c-l) | o | x |
|  | 37 | 1b | 1b | 6 (c-l) | o | x |
|  | 42 | 1b | 1b | 6 (c-l) | o | x |
|  | 48 | 1b | 1b | 1a | o | x |
|  | 51 | 1b | 1b | 1a | o | x |
|  | 54 | 1b | 1b | 6 (c-l) | o | x |
|  | 55 | 6i | 6i or 6n | 1b | o | x |
|  | 56 | 1b | 1b | 6 (c-l) | o | x |
|  | 70 | No data | 1b | 1a | - | - |
|  | 74 | 6i | 6i or 6n | 1b | o | x |
|  | 77 | 1b | 1b | 6 (c-l) | o | x |
|  | 79 | 1b | 1b | 6 (c-l) | o | x |
|  | 90 | 1b | 1b | 6 (c-l) | o | x |
|  | 92 | 6i | 6i or 6n | 1b | o | x |
|  | 93 | 6i | 6i or 6n | 1b | o | x |
|  | 95 | 1b | 1b | 6 (c-l) | o | x |
|  | 98 | 1b | 1b | 6 (c-l) | o | x |
|  | 100 | 1b | 1b | 6 (c-l) | o | x |
|  | 101 | negative | Negative | 6 (c-l) | o | x |
|  | 106 | 6i | 6i or 6n | 1a | o | x |
|  | 107 | 1b | 1b | 6 (c-l) | o | x |
|  | 116 | 1b | 1b | 6 (c-l) | o | x |
|  | 118 | 1b | 1a | 6 (c-l) | x | x |
|  | 124 | 6i | 6i or 6n | 1b | o | x |
|  | 127 | No data | 1b | 1a | - | - |
|  | 132 | 6i | 6i or 6n | 1a | o | x |
|  | 134 | 6i | 6i or 6n | 1b | o | x |
|  | 136 | 1b | 1b | 1a | o | x |
|  | 148 | 6f | 3 & 6a or 6f | 3 | o | x |
|  | 150 | No data | 1b | 1a | - | - |
|  | 151 | 6i | 6i or 6n | 1b | o | x |
|  | 161 | 1b | 1b | 6 (c-l) | o | x |
|  | 181 | 6i | 6i or 6n | 1b | o | x |
|  | 182 | No data | 1a | 6 (c-l) | - | - |
|  | 187 | 1b | 1b | 6 (c-l) | o | x |

x- miss-matched with sequencing; o- matched with sequencing

**Table S2:** TP, TN, FP, and FN results of 6 HCV Genotyping 9G Test and LiPA 2.0 with the sequencing in 274 samples

| **HCV genotype**  **(Test)** | | **TP** | **TN** | **FP** | **FN** |
| --- | --- | --- | --- | --- | --- |
| **1a** | 6 HCV Genotyping 9G test | 22 | 251 | 1 | 0 |
|  | LiPA 2.0 Assay | 22 | 244 | 8 | 0 |
| **1b** | 6 HCV Genotyping 9G test | 56 | 217 | 0 | 1 |
|  | LiPA 2.0 Assay | 33 | 207 | 10 | 24 |
| **2** | 6 HCV Genotyping 9G test | 3 | 271 | 0 | 0 |
|  | LiPA 2.0 Assay | 3 | 271 | 0 | 0 |
| **3** | 6 HCV Genotyping 9G test | 60 | 213 | 0 | 0 |
|  | LiPA 2.0 Assay | 60 | 212 | 1 | 0 |
| **4** | 6 HCV Genotyping 9G test | 0 | 274 | 0 | 0 |
|  | LiPA 2.0 Assay | 0 | 274 | 0 | 0 |
| **6** | 6 HCV Genotyping 9G test | 49 | 226 | 0 | 0 |
|  | LiPA 2.0 Assay | 35 | 206 | 20 | 13 |
| **Negative** | 6 HCV Genotyping 9G test | 192 | 82 | 0 | 0 |
|  | LiPA 2.0 Assay | 192 | 80 | 2 | 0 |

**Table S3:** TP, TN, FP, and FN results of 6 HCV Genotyping 9G Test (n=274)

| **Test** | **TP** | **TN** | **FP** | **FN** |
| --- | --- | --- | --- | --- |
| **6 HCV Genotyping 9G test** | **190** | **82** | **1** | **1** |
